# Integration of Physiological, Transcriptomic, and Metabolomic Analyses Reveal Molecular Mechanisms of Salt Stress in *Maclura tricuspidata*

**DOI:** 10.3390/plants13030397

**Published:** 2024-01-29

**Authors:** Dezong Sui, Baosong Wang, Yousry A. El-Kassaby, Lei Wang

**Affiliations:** 1 Jiangsu Academy of Forestry, Nanjing 211153, China; jslky@jaf.ac.cn (D.S.); baoswang@163.com (B.W.); 2 Department of Forest and Conservation Sciences, Faculty of Forestry, University of British Columbia, Vancouver, BC V6T IZ4, Canada; y.el-kassaby@ubc.ca

**Keywords:** *Maclura tricuspidate*, resistance, salt adaptation, multi-omics

## Abstract

Salt stress is a universal abiotic stress that severely affects plant growth and development. Understanding the mechanisms of *Maclura tricuspidate*’s adaptation to salt stress is crucial for developing salt-tolerant plant varieties. This article discusses the integration of physiology, transcriptome, and metabolome to investigate the mechanism of salt adaptation in *M. tricuspidata* under salt stress conditions. Overall, the antioxidant enzyme system (SOD and POD) of *M. tricuspidata* exhibited higher activities compared with the control, while the content of soluble sugar and concentrations of chlorophyll a and b were maintained during salt stress. KEGG analysis revealed that deferentially expressed genes were primarily involved in plant hormone signal transduction, phenylpropanoid and flavonoid biosynthesis, alkaloids, and MAPK signaling pathways. Differential metabolites were enriched in amino acid metabolism, the biosynthesis of plant hormones, butanoate, and 2-oxocarboxylic acid metabolism. Interestingly, glycine, serine, and threonine metabolism were found to be important both in the metabolome and transcriptome–metabolome correlation analyses, suggesting their essential role in enhancing the salt tolerance of *M. tricuspidata*. Collectively, our study not only revealed the molecular mechanism of salt tolerance in *M. tricuspidata*, but also provided a new perspective for future salt-tolerant breeding and improvement in salt land for this species.

## 1. Introduction

Soil salinization is a major global environmental and socioeconomic issue. Currently, approximately 1.125 × 10^9^ ha of saline–alkali soil exists worldwide [1]. By 2050, it is estimated that over half of the world’s arable land will be affected by salinization [2,3]. The total area of salinized and alkaline land in China is widespread, reaching approximately 5.0 × 10^8^ ha [4]. Salt stress caused by neutral salts, such as NaCl and Na_2_SO_4_, can lead to water deficiency, ion toxicity, decreased nutrient absorption and transport in plant bodies, as well as the accumulation of reactive oxygen species (ROS) that can be toxic to cells [5]. Saline–alkali soil adversely affects plant growth and development, severely restricting the development of sustainable agriculture [6].

Plants develop a variety of physiological and biochemical adaptive mechanisms in response to soil salt stress, known as the plant salt stress response [7]. These mechanisms primarily revolve around regulating ion, osmotic, and nutrient balances, and scavenging ROS [8]. Collectively, these processes enable plants to adapt and survive in environments with high salt concentrations. Studies have shown significant changes in plants in response to salt stress, including oxidative and ion balances and osmotic adjustment [9,10]. ROS is vitally important in responding to stress perception, integrating various stress response signaling networks, activating and domesticating plant defense mechanisms [11]. Under salt stress, plants have the ability to activate enzymatic and non-enzymatic systems to protect cells from ROS toxicity [12,13]. The enzymatic system is composed of antioxidant enzymes, such as catalase (CAT), peroxidase (POD), and superoxide dismutase (SOD) [14]. Non-enzymatic systems generate metabolites that help to eliminate ROS from cells during salt stress, such as ascorbic acid, alkaloids, carotenoids, flavonoids, glutathione, phenolic compounds, and melatonin [7,14,15,16].

Technological advancements and innovation enable the understanding of changes in genes and metabolites within organisms [17]. High-throughput technologies have facilitated the study of molecular mechanisms and metabolic regulation pathways in plants [18,19]. Although salt tolerance has been proven in certain species, current research primarily centers on model plant species (e.g., *Arabidopsis thaliana*, *Solanum lycopersicum*, *Oryza sativa*, *Triticum aestivum*, and *Populus alba × P. glandulosa*) [20,21,22,23,24]. Therefore, it is essential to explore the molecular regulatory mechanisms of plant responses to salt stress, meanwhile, further exploration is needed for the development and utilization of salt-tolerant plant resources.

*Maclura tricuspidata* (Carr.) Bur. ex Lavallee, Moraceae, is a deciduous broad-leaved spiny tree distributed throughout China, Korea, and Japan [25]. *M. tricuspidata* is widely used in folk medicine to treat various disorders [26]. Additionally, bark fibers are used for papermaking, leaves serve as silkworms feed, and its fruit is consumed [27]. *M. tricuspidata* is a multi-purpose tree species that can survive on moderately saline–alkali soils. In the present study, we employed a combination of multi-omics techniques in *M. tricuspidata* to: (1) elucidate the effects of salt stress on genes and metabolites; (2) identify the key biological pathways associated with regulating salt tolerance; (3) explore the regulatory network of gene–metabolite underlying salt tolerance; and (4) elaborate the molecular mechanisms of salt adaptation. Our study systematically investigated the adaptive mechanisms of *M. tricuspidata* leaves in response to salt stress from three aspects: physiology, transcriptomics, and metabolomics, which are expected to provide new insights into the salt stress adaptation mechanisms of *M. tricuspidata*.

## 2. Results

### 2.1. Effects of Salt Stress on Plant Phenotypic Differences and Physiological Characteristics

**Figure 1:**
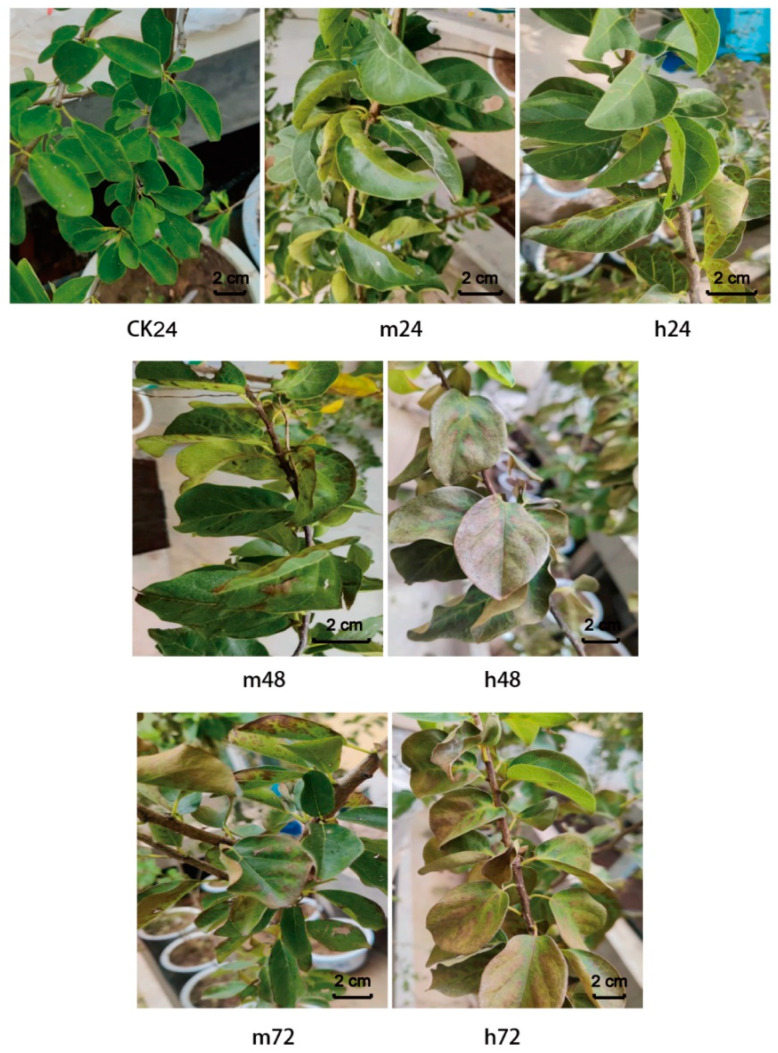
Phenotypic differences of *M. tricuspidata* leaves under control (CK), moderate (m), and high (h) salt stress treatments for 24, 48, and 72 h, respectively. Note: CK, 0 mM NaCl; m24, 68 mM NaCl treatment for 24 h; h24, 102 mM NaCl treatment for 24 h; m48, 68 mM NaCl treatment for 48 h; h48, 102 mM NaCl treatment for 48 h; m72, 68 mM NaCl treatment for 72 h; h72, 102 mM NaCl treatment for 72 h.

Compared with the control treatment without salt (CK), *M. tricuspidata* treated with high (h) salt concentration (102 mM NaCl) for 24 h exhibited a few necrotic spot symptoms on the upper surface of the leaves (Figure 1). Furthermore, leaves treated for 48 and 72 h showed symptoms of larger, clearly visible necrotic patches. These symptoms were more obvious under the h treatment than the moderate (m) treatment (68 mM NaCl).

**Figure 2 plants-13-00397-f002:**
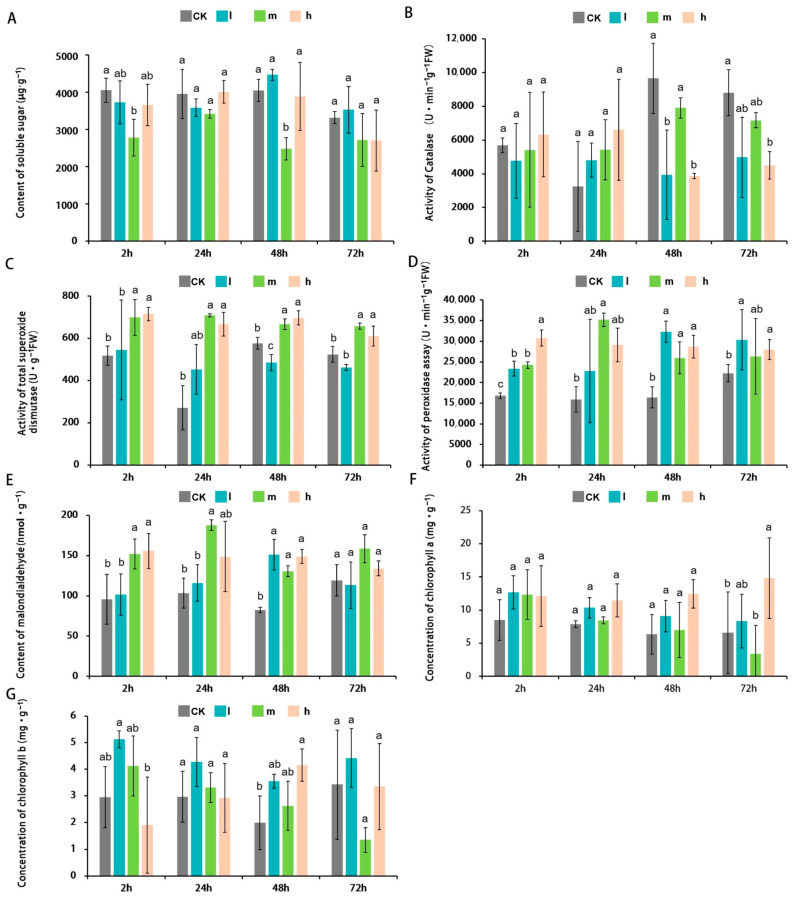
Physiological characteristics of *M. tricuspidata* under salt stress. (**A**) Content of soluble sugar. (**B**–**D**) Activities of CAT, SOD, and POD. (**E**) Content of MDA. (**F**,**G**) Concentration of chlorophyll a and b. CK, 0 mM NaCl; l, 34 mM NaCl; m, 68 mM NaCl; h, 102 mM NaCl. Different letters indicate a statistically significant difference when analyzed by one-way ANOVA and a multiple comparisons using Duncan’s method at *p* ≤ 0.05.

Salt stress can disrupt the balance of ROS scavenging in plants, leading to oxidative stress reactions [28]. Therefore, physiological assessments were conducted to examine the characteristics of *M. tricuspidata* leaves under salt stress. The results showed that there were no significant changes in the levels of soluble sugar among the three different salt stress treatments at the four time points ([Fig plants-13-00397-f002]A). CAT activities began to exhibit a significant decrease under 48 h (Figure 2B). SOD activities showed an overall increasing trend, especially under the m and h treatments, with a significant difference compared with the CK (Figure 2C). POD activities significantly increased during most stress treatments, except for 24 h under low-concentration (l) and h treatments, and 72 h under the m treatment (Figure 2D). In comparison to the CK, the MDA content significantly increased under the m and h treatments at 2 h, the m treatment at 24 h, and all three treatments at 48 h (Figure 2E). The concentrations of chlorophyll a and b did not show significant differences among the various treatments (Figure 2F,G). Overall, under salt stress conditions, *M. tricuspidate* was able to maintain an appropriate level of soluble sugar content, and exhibited relatively higher activities of SOD, and POD. Despite experiencing membrane lipid peroxidation, *M. tricuspidate* still maintained stable concentrations of chlorophyll a and b. This physiological adaptation ability may contribute to *M. tricuspidate*’s resistance against salt stress.

### 2.2. Summary of Transcriptome Sequencing Data

In total, 164.79 Gb of raw data was generated, and the average GC content was 45.52% (Supplementary Table S1). The sequencing quality achieved Q20 and Q30 scores of 97.47 and 92.96%, respectively. PCA results indicated that the biological replicates within each group were clustered together (Supplementary Figure S1A). Moreover, the correlation among samples showed good reproducibility between the biological replicates, which was consistent with the PCA results, indicating a high level of reproducibility (Supplementary Figure S1B).

### 2.3. Screening and Identification of Differentially Expressed Genes 

**Figure 3:**
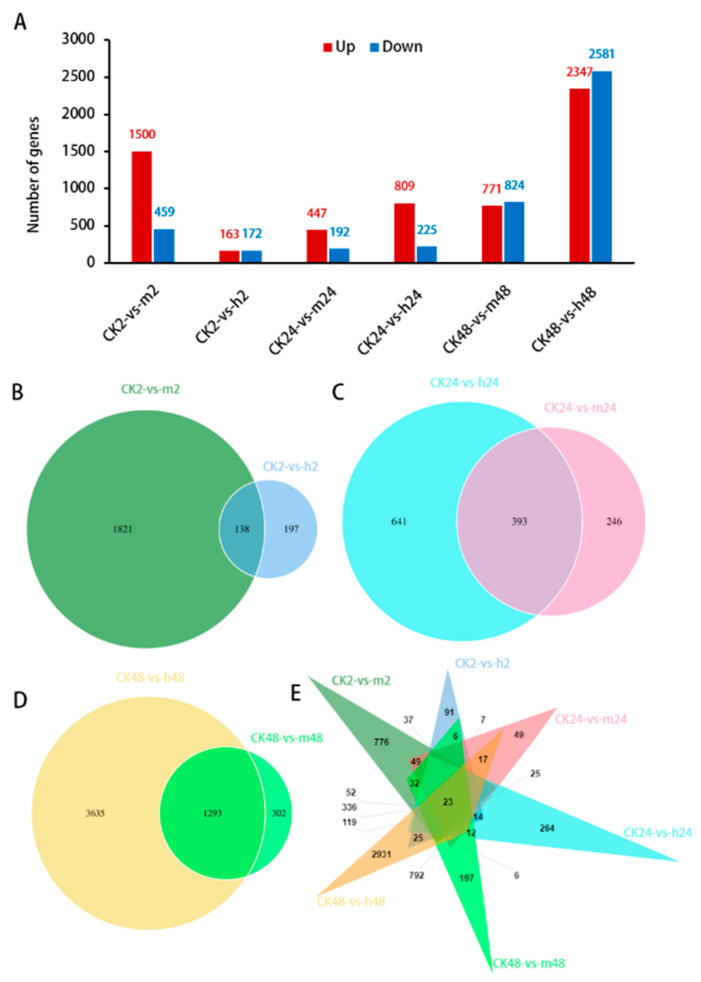
The number of DEGs in different comparison groups (CK2-vs.-m2, CK2-vs.-h2, CK24-vs.-m24, CK24-vs.-h24, CK48-vs.-m48, and CK48-vs.-h48). (**A**) Number of up and down DEGs in six comparison groups. (**B**) Common DEGs under two comparison groups under 2 h treatment. (**C**) Common DEGs under two comparison groups under 24 h treatment. (**D**) Common DEGs under two comparison groups under 48 h treatment. (**E**) Common DEGs among six comparison groups.

The present study specifically focused on the analysis of differentially expressed genes (DEGs) in six comparison groups. In the early stages of salt stress (2 h and 24 h) (Figure 3A), the number of up-regulated genes was significantly higher than that of down-regulated genes in four comparison groups, except for the comparison of CK2-vs.-h2, where the number of up-regulated and down-regulated genes were similar. Interestingly, the number of DEGs under the h treatment was lower than that under the m treatment at 2 h, while the number of DEGs under the h treatment was higher than that under the m treatment at 24 h. Until 48 h of stress treatment, the number of DEGs increased significantly, particularly under the h treatment, where the number of DEGs showed a sharp increasing trend. Thus, the salt stress response genes in *M. tricuspidata* were significantly expressed after 48 h of salt stress. In addition, under two different concentrations of salt stress (m and h) at the same treatment time (2, 24, and 48 h), a total of 138, 393, and 1293 commonly DEGs were identified, respectively (Figure 3B–D). Among these, 23 DEGs were found to be common to all six comparison groups (Figure 3E).

### 2.4. Gene Expression Validation by qRT-PCR

**Figure 4:**
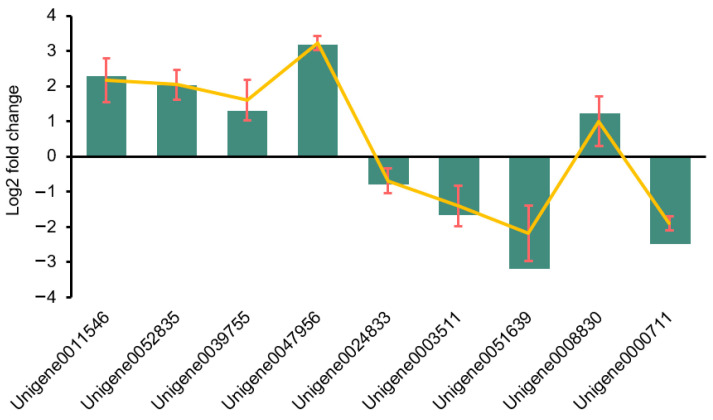
Relative gene expression of DEGs from RNA-seq data verified by qRT-PCR. Note: The green histogram indicates the value of FPKM from RNA-seq and the yellow line indicates the expression level of nine genes detected by qRT-PCR; the error line represents SD calculated from three replicates of qRT-PCR.

Nine DEGs in the CK48-vs.-h48 comparison were randomly selected for qRT-PCR validation (Figure 4). The expression patterns of these nine genes were consistent with the transcriptomic data, with a correlation coefficient (R) of 0.9932, suggesting that the gene expression patterns detected by RNA-seq are reliable.

### 2.5. Functional Annotation Analysis of DEGs

**Figure 5:**
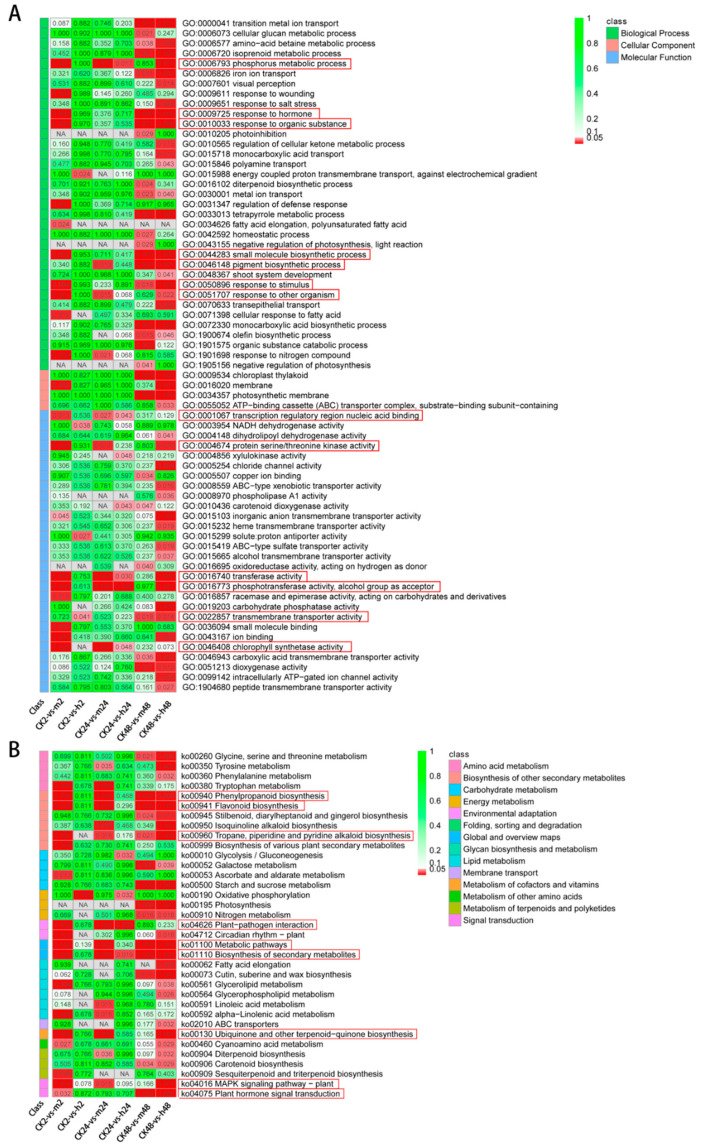
Enrichment analysis of DEGs in response to salt stress. (**A**) GO functional annotation of DEGs. (**B**) Enrichment analysis of DEGs in KEGG pathways. Darker red color indicates higher degree of enrichment. NA is referred to No Abundance.

To understand the functions of DEGs, GO and KEGG enrichment analysis were performed. The results revealed that 13 GO terms from at least three groups were significantly enriched in six different comparisons (Figure 5A). These terms included phosphorus metabolic process (GO:0006793), response to hormone (GO:0009725), response to organic substance (GO:0010033), small molecule biosynthetic process (GO:0044283), pigment biosynthetic process (GO:0046148), response to stimulus (GO:0050896), and response to other organism (GO:0051707) in biological process ontology. Additionally, transcription regulatory region nucleic acid binding (GO:0001067), protein serine/threonine kinase activity (GO:0004674), transferase activity (GO:0016740), phosphotransferase activity, alcohol group as acceptor (GO:0016773), transmembrane transporter activity (GO:0022857), and chlorophyll synthetase activity (GO:0046408) were identified in the molecular function ontology. Moreover, KEGG enrichment results showed that 9 pathways from at least three groups were significantly enriched (Figure 5B). These pathways included phenylpropanoid biosynthesis (ko00940), flavonoid biosynthesis (ko00941), tropane, piperidine and pyridine alkaloid biosynthesis (ko00960), plant–pathogen interaction (ko04626), metabolic pathways (ko01100), biosynthesis of secondary metabolites (ko01110), ubiquinone and other terpenoid-quinone biosynthesis (ko00130), mitogen-activated protein kinase (MAPK) signaling pathway-plant (ko04016), and plant hormone signal transduction (ko04075).

#### 2.5.1. Plant Hormone Signal Transduction

**Figure 6:**
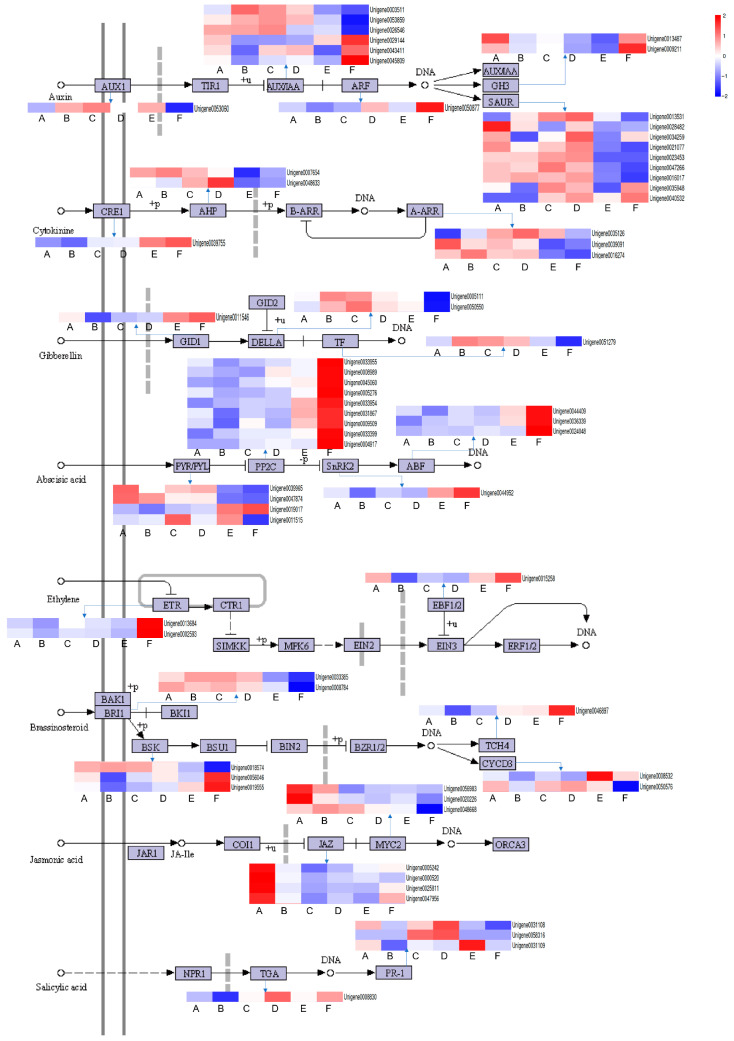
Expression of DEGs involved in the plant hormone signal transduction pathway of *M. tricuspidata* under salt stress. (A) The comparison of CK2-vs.-m2. (B) CK2-vs.-h2. (C) CK24-vs.-m24. (D) CK24-vs.-h24. (E) CK48-vs.-m48. (F) CK48-vs.-h48. The redder the heatmap color, the higher the level of gene expression.

Plant hormones play a critical role in plant responses to environmental stresses. In this study, we found that the plant hormone signal transduction pathway was significantly enriched in three comparison groups. Specifically, auxin influx carrier (*AUX1*), auxin/indole-3-acetic acid (*AUX/IAA*), and auxin response factor (*ARF*), which are involved in auxin signaling, were more significantly upregulated under salt stress (Figure 6). Gretchen hagen3 (*GH3*), an early responsive genes of plant auxin, was upregulated only in the first (CK2-vs.-m2) and sixth (CK48-vs.-h48) comparison groups. Small auxin-up RNAs (*SAUR*), another early responsive gene of plant auxin, was quickly and specifically upregulated after salt stress treatment. In the cytokinine (CTK) signal transduction pathway, cytokinin receptor (*CRE1*) was significantly upregulated under 48 h treatment, and *AHP* and *ARR* were found to be upregulated in various comparison groups. In the gibberellin (GA) signaling pathway, gibberellin-insensitive dwarf 1 (*GID1*) and *DELLA* were significantly upregulated in various comparison groups. In contrast, in the abscisic acid (ABA) pathway, pyrabactin resistance/-likes (*PYR*/*PYL*), protein phosphatase type Ⅱ C (*PP2C*), sucrose non-fermenting 1-related protein kinase 2 (*SnRK2*), and ABA responsive element binding factor (*ABF*), which are the main proteins involved in the ABA receptor signaling pathway, exhibited a downward trend in expression in most comparison groups, except for nine *PP2C*, one *SnRK2*, and three *ABF*, which were upregulated in the CK48-vs.-h48 comparison. In the ethylene pathway, ethylene receptor (*ETR*) and EIN3-binding F box protein 1 and 2 (*EBF1/2*) were mainly downregulated, except for CK48-vs.-h48. In the brassinosteroid (BR) pathway, BR insensitive 1 (*BRI1*), BR signaling kinase (*BSK*), touch 4 (*TCH4*), and D-type cyclin 3 (*CYCD3*) were upregulated in various comparison groups. In the jasmonic acid (JA) pathway, jasmonate ZIM domain-containing protein (*JAZ*) and myelocytomatosis protein 2 (*MYC2*) were downregulated in several comparison groups but significantly upregulated under CK2-vs.-m2. Finally, one DEG encoding the transcription factor *TGA* and three pathogenesis-related protein 1 (*PR-1*) in the salicylic acid (SA) signaling pathway were downregulated in the early treatment (2 h) but upregulated in the 24 and 48 h treatments.

#### 2.5.2. Phenylpropanoid Biosynthesis

**Figure 7:**
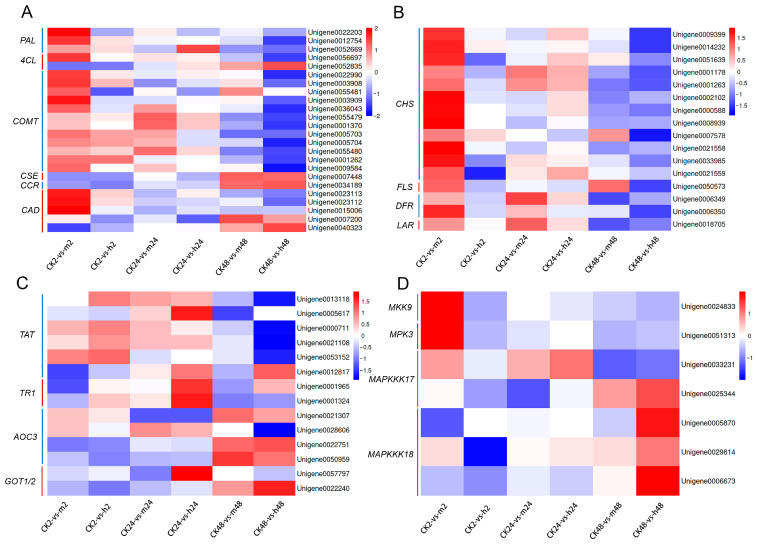
Expression of DEGs in four important metabolic pathways under salt stress. (**A**) Phenylpropanoid biosynthesis. (**B**) Flavonoid biosynthesis. (**C**) Tropane, piperidine and pyridine alkaloid biosynthesis. (**D**) MAPK signaling pathway plant. The redder the heatmap color, the higher the level of gene expression.

Phenylpropanoids play an important role in plants’ response to both biotic and abiotic stresses. In our study, we observed that DEGs involved in the phenylpropanoid biosynthesis pathway were significantly affected by salt stress treatment across six comparison groups (Figure 7A). Key enzymes in the phenylpropanoid biosynthesis pathway, including phenylalanine ammonialyase (*PAL*), 4-coumarate-CoA ligase (*4CL*), caffeate/5-hydroxyferulate 3-O-methyltransferase (*COMT*), caffeoyl shikimate esterase (*CSE*), cinnamoyl-CoA reductase (*CCR*), and cinnamyl alcohol dehydrogenase (*CAD*), exhibited differential expression patterns among the six comparison groups, particularly in the CK2-vs.-m2 group, where most of these enzymes showed an upregulated expression. Interestingly, the expression trend of these enzymes in the CK48-vs.-h48 group was opposite to that in the CK2-vs.-m2 group. However, it is worth noting that, as the salt concentration increased, the change in gene expression at the same treatment time became more consistent.

#### 2.5.3. Flavonoid Biosynthesis

Genes involved in flavonoid biosynthesis, including chalcone synthase (*CHS*), flavonol synthase (*FLS*), dihydroflavonol 4-reductase (*DFR*), and leucoanthocyanidin reductase (*LAR*), were significantly enriched in all six comparison groups (Figure 7B). Interestingly, these four genes exhibited completely opposite expression patterns between the CK2-vs.-m2 (upregulated change) group and the CK48-vs.-h48 group (downregulated change).

#### 2.5.4. Tropane, Piperidine, and Pyridine Alkaloid Biosynthesis

In the tropane, piperidine, and pyridine alkaloid biosynthesis pathway, four types of gene transcripts were identified as tyrosine aminotransferase (*TAT*), tropinone reductase 1 (*TR1*), allene oxide cyelase 3 (*AOC3*), and glutamic-oxaloacetic transaminase (*GOT1/2*). Most of these genes exhibited upregulated expression levels across the six comparison groups (Figure 7C). 

#### 2.5.5. MAPK Signaling Pathway Plant

MAPK signaling pathway showed significant enrichments in all six comparison groups (Figure 7D). This pathway included important genes such as mitogen-activated protein kinase kinase (*MKK9*), mitogen-activated protein kinase (*MPK3*), and mitogen-activated protein kinase kinase kinase (*MAPKKK17*/*18*). In the early stage of treatment, *MKK9* and *MPK3* showed upregulation, but as the concentration and time increased, their expression levels decreased. Conversely, one *MAPKKK17* and three *MAPKKK18* genes displayed downregulation in the early stage, but their expression levels increased with a higher concentration and longer treatment time.

### 2.6. Summary of the LC-MS/MS Metabolomics

In the current study, both positive and negative ion mode (POS and NEG) were employed to achieve a wider coverage of metabolites and obtain more desirable detection results. A total of 1506 and 1721 known metabolites were successfully identified under the POS and NEG modes, respectively. The number of differentially accumulated metabolites (DAMs) is shown in Table 1.

### 2.7. Differential Metabolites Respond to Salt Stress

**Figure 8:**
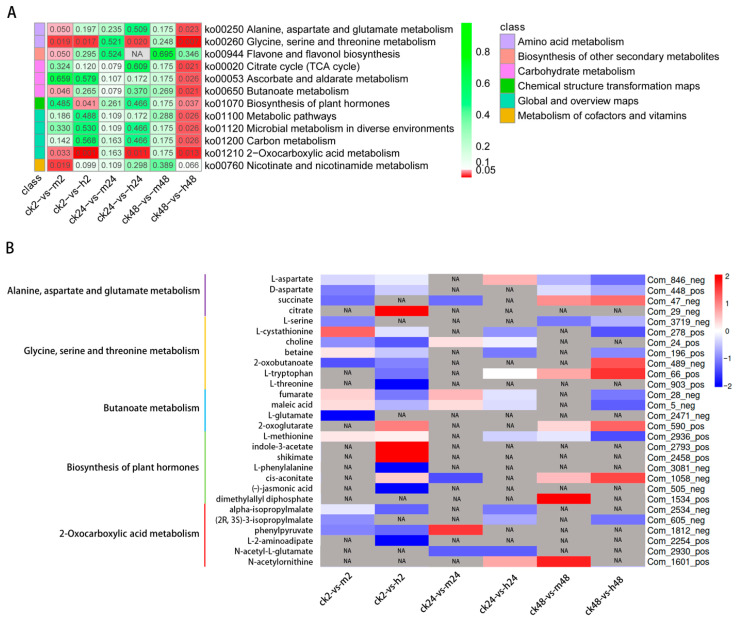
Differential metabolites of *M. tricuspidata* in response to salt stress between different comparisons: (**A**) KEGG analysis of differential metabolites; and (**B**) Abundance of DAMs in five pathways. NA is referred to No Abundance.

The KEGG pathway enrichment analysis revealed a significant enrichment of several pathways including alanine, aspartate and glutamate metabolism (ko00250), glycine, serine, and threonine metabolism (ko00260), butanoate metabolism (ko00650), biosynthesis of plant hormones (ko01070), and 2-oxocarboxylic acid metabolism (ko01210) in at least two comparison groups. Among these, the pathway of glycine, serine, and threonine metabolism and 2-oxocarboxylic acid metabolism were significantly enriched in four comparison groups (Figure 8A). Consequently, the subsequent analysis focuses on the detailed exploration of these five pathways (Figure 8B).

#### 2.7.1. Alanine, Aspartate, and Glutamate Metabolism

Across the six comparison groups under stress treatment, four differential metabolites (L-aspartate, D-aspartate, succinate, and citrate) were found to be significantly enriched in the alanine, aspartate, and glutamate metabolism pathway. Among these metabolites, L-aspartate exhibited a significant increase within 24 h under high concentration stress, succinate showed a significant increase within 48 h under stress, and citrate displayed a significant increase within 2 h under the h treatment. However, the metabolites in other parts either significantly decreased or were not detected.

#### 2.7.2. Glycine, Serine, and Threonine Metabolism

Among the six comparison groups analyzed, seven metabolites (L-serine, L-cystathionine, choline, betaine, 2-oxobutanoate, L-tryptophan, and L-threonine) involved in glycine, serine, and threonine metabolism were found to be enriched in four comparison groups. Specifically, at three time points under the h treatment, L-cystathionine under the m treatment for 2 h, 2-oxobutanoate under the h treatment for 48 h, and L-tryptophan for 48 h, the abundance of these metabolites significantly increased during these specific treatment conditions.

#### 2.7.3. Butanoate Metabolism

Four metabolites (fumarate, maleic acid, L-glutamate, and 2-oxoglutarate) involved in butanoate metabolism were found to be significantly enriched in the first and sixth comparison groups. Fumarate and maleic acid showed a slight increase at 2 h and 24 h under the m treatment, while the abundance of 2-oxoglutarate increased under the h treatment at 2 h and 48 h.

#### 2.7.4. Biosynthesis of Plant Hormones

Plant hormones play a crucial role in regulating various metabolic processes to adapt to changes in external environmental conditions. Seven metabolites (L-methionine, indole-3-acetate, shikimate, L-phenylalanine, cis-aconitate, (−)-jasmonic acid, and dimethylallyl diphosphate) involved in the biosynthesis of plant hormone pathways were significantly enriched under the h treatment at 2 h and 48 h. The abundance of indole-3-acetate and shikimate significantly increased under the h treatment at 2 h. Cis-aconitate abundance increased under the h treatment at 2 h, as well as under the m and h treatments at 48 h. Additionally, the abundance of dimethylallyl diphosphate significantly increased under the m treatment at 48 h.

#### 2.7.5. 2-Oxocarboxylic Acid Metabolism

Six metabolites (alpha-isopropylmalate, (2R,3S)-3-isopropylmalate, phenylpyruvate, L-2-aminoadipate, N-acetyl-L-glutamate, and N-acetylornithine) involved in 2-oxycarboxylic acid metabolism were significantly enriched in four out of the six comparison groups, including the m and h treatment at 2 h, and the h treatment at 24 h and 48 h. Phenylpyruvate abundance showed an increasing trend under the m at 24 h, while N-acetylornithine abundance increased under the h treatment at 24 h and the m treatment at 48 h.

### 2.8. Conjoint Analysis of Transcriptomic and Metabolomic Data

The establishment and visualization of gene–metabolite interactions were based on KEGG pathway maps. We conducted a comprehensive analysis of omics data under 2, 24, and 48 h of salt treatment, resulting in the identification of 10,388 DEGs and 650 DAMs across all comparisons. Two pathways, namely metabolic pathways, glycine, serine, and threonine metabolism, were found to be highly significantly enriched in the sixth comparison groups (Supplementary Tables S2 and S3). Furthermore, the glycine, serine, and threonine metabolism pathway, which was observed in both the metabolome and transcriptome–metabolome correlation analyses, may be the essential biological processes in response to salinity.

**Figure 9:**
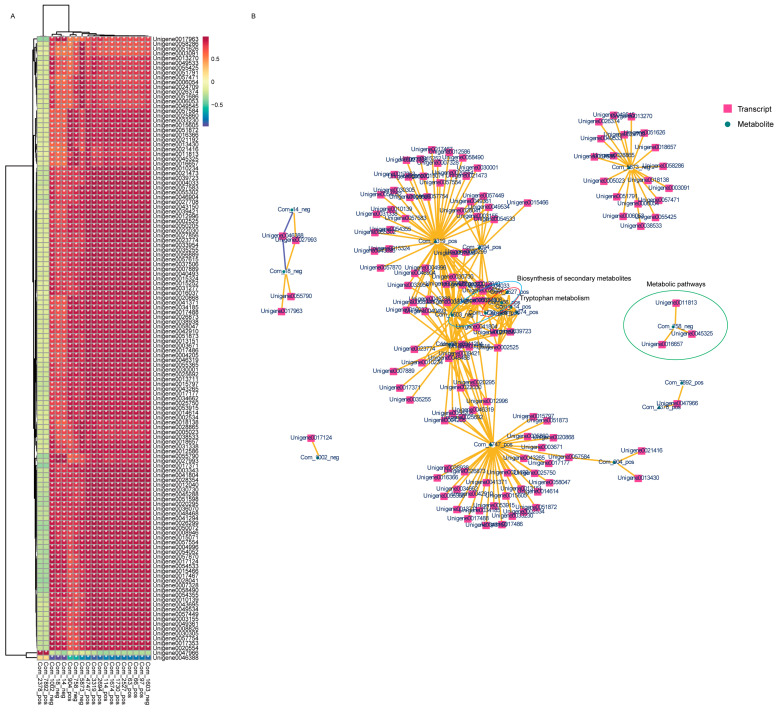
Correlation analysis of DEGs and DAMs in *M. tricuspidate* under salt stress. (**A**) Heatmap of the top 250 DEGs and 19 DAMs. (**B**) Cytoscape network of the top DEGs and top DAMs within the same KEGG pathways. The asterisk (*) represents positive correlation.

Pearson correlation coefficient was used to evaluate the relationship between DEGs and DMAs. Ultimately, the top 250 DEGs were selected based on their correlation coefficients with DMAs. The expression heatmap of these DEGs and DMAs was shown in Figure 9A. It can be observed that the DMA of Com_2378_pos and Com_7892_pos were significantly positively correlated with other DEGs (except Unigene0047966), while Unigene0047966 was positively correlated with other DMAs, and Unigene0046388 was positively correlated with all DMAs. Moreover, a network diagram of the correlation coefficients between these DEGs and DMAs is presented in Figure 9B. The biosynthesis of secondary metabolites, tryptophan metabolism, and metabolic pathways were prominent, indicating that these three pathways may play a pivotal part in the transcriptional and metabolic response to salt stress.

## 3. Discussion

### 3.1. Salinity-Activated Hormone Signaling to Participate in Stress Response

Plants optimize the balance between growth and stress by integrating exogenous salt stress and endogenous development signals [29,30]. Generally, significant changes occurring in plants under stress conditions can affect hormone flux and signal transduction [31].

Auxin participates in plants’ adaptation to environmental stress by regulating developmental processes and morphological structures of plant leaves [32]. Salt stress commonly leads to a reduction in IAA levels and alters the localization of *AUX1* and *Pin1*/*2* [33], resulting in the downregulation of auxin receptor-encoding genes *TIR1* and *AFB2*, inhibiting plant growth [34]. Our transcriptome analysis revealed that the gene expression of *AUX1*, *AUX*/*IAA*, and *SAUR* in the IAA signal transduction pathway was mostly upregulated initially, but started to downregulate under 48 h of stress treatment. This suggests that the initial salt stress may promote the auxin signal transduction process but inhibits it during the later stages of stress. In cotton, an increase in IAA levels and the activation of the auxin signal transduction pathway contribute to salt stress adaptation [35]. Similarly, in *Arabidopsis thaliana*, an increase in IAA levels also enhances salt tolerance [36]. These findings are consistent with our current results, indicating that salinity activates the transcription of key genes involved in IAA signal transduction and enhances salt adaptation.

The appropriate modulation of CTK metabolism and signal transduction are beneficial for plant survival in saline environments [37]. Under salt stress conditions, the expression of CTK signal components, such as *AHK*, *AHP*, *CRF*, and *ARR*, undergoes significant changes [29]. In the ‘84 K’ poplar clone, salt stress was found to activate the cytokinin signaling pathway, and 59 *ARRs* showed transcriptional changes during salt treatment, indicating their potential involvement in salt response [38]. Consistent with these findings, we observed that the transcription of *AHP* and *A-ARR* was inhibited in the later stage of the CTK signaling pathway under salt stress. However, *CRE1* exhibited an upregulated expression pattern.

The dynamic balance mechanism of active GA content in plants relies on the core components of the GA signaling pathway, including DELLA proteins (such as RGA, GAI, RGL1/2/3), F-box protein SLEEPY1, and soluble GA receptor protein GID1 [39,40]. When exposed to salt stress, *Arabidopsis* experiences a decrease in GA content, while the accumulation of DELLA proteins can enhance plant resistance to salt stress [41]. Additionally, DELLA proteins can counteract the production of ROS induced by abiotic stress, thereby increasing the plant’s resilience [42]. In our observations, we observed an increase in *GID1* expression level under 48 h treatment, while *DELLA* showed the opposite trend.

ABA plays an essential role in defending against salt stress [43,44]. Under salt stress, ABA activates downstream response genes, forming a PYL–ABA–PP2C complex that removes the PP2C inhibition of SnRK2. This leads to SnRK2 phosphorylating TFs, which helps maintain ion balance and clears ROS, thereby mitigating the damage caused by stress [8]. In the ‘84 K’ poplar, researchers constructed a subnetwork primarily consisting of *ABF* and *PP2C* in the ABA transduction pathway [38]. In our study, we observed the upregulation of four different *PYL* genes in different comparison groups, but the expression levels of *PP2C*, *SnRK2*, and *ABF* were the highest under 48 h of stress treatment. These results differ from those found by Zhao et al. [38], which might be attributed to the fact that they studied six different time points in poplar, whereas we only examined three time points in our study.

Ethylene signal transduction plays an important role in plant’s response to salt stress [29]. The exogenous application of ethylene increased the sensitivity of rice seedlings to salt, while the application of the ethylene inhibitor 1-methylcyclopropene (1-MCP) could enhance rice’s tolerance to salt [45]. The lectin receptor-like kinase *OsSIT1* activated *MPK3*/*6*, leading to increased ethylene biosynthesis, accumulation of ROS, and salt sensitivity [46]. 

In *Arabidopsis* subjected to high salt treatment, the degradation of two F-box proteins, EBF1/ EBF 2, was promoted, facilitating the accumulation of two core TFs, *EIN3* and *EIL1* [47]. However, our study found that the expression of *ETR* and *EBF1*/*2* genes was upregulated after 48 h of high salt stress, which is inconsistent with the findings of Peng et al. [47]. This discrepancy suggests that the different regulatory mechanisms may exist in various plant species.

BRs play a vital role in regulating plant salt tolerance; however, their effect is not always positive. Liu et al. (2020) proposed that appropriately increasing the level or enhancing the signaling of BRs can improve plant salt tolerance, but excessive or insufficient levels of BRs may be detrimental to plant salt tolerance [48]. The membrane-bound receptor *BRI1* recognizes the presence of BR, inducing the formation of a heterodimer between *BRI1* and BRI1-associated kinase 1 (*BAK1*), which in turn activates downstream components such as *BSKs* and BR-insensitive 2 (*BIN2*), ultimately leading to the activation of BR-responsive genes [49]. *ZmBES1*/*BZR1-5* has been reported to have increased expression in maize shoots, while its expression is consistently suppressed in roots, indicating that salt stress may activate distinct BR responses in different tissues/organs within the same plant [50]. Our research found that the expression pattern of one of the three *BSK* genes was consistent with that of *BRI1*, and the expression levels of these genes increased at 2 h and 24 h of salt treatment, indicating that the *BRI1* gene plays a regulatory role in downstream *BSK* genes.

JA is one of the key hormones involved in plant responses to abiotic stress. Previous studies have shown that JA enhances tomato’s salt tolerance by maintaining the balance of ROS [21]. JAZ proteins act as transcriptional repressors, inhibiting the activity of key TFs (such as basic helix–loop–helix (*bHLH*), *MYC2*, and its homologues *MYC3*/*4*/*5*) in the JA pathway [51]. Herein, we observed that the expression of *JAZ* and the major regulatory factor *MYC2* was upregulated in the early stages of salt stress and downregulated in the later stages in the JA signaling pathway, indicating that salt stress activates JA signal transduction.

SA, as a key defensive hormone, can alleviate salt stress in plants [52]. SA enhances the antioxidant system, promotes the synthesis of osmotic substances, and accelerates plant photosynthesis under salt stress [53]. The transcriptomic analysis of cotton has shown that important *TGA* elements involved in the SA signaling pathway were downregulated [35]. In this study, we found that the expression levels of TGA genes decreased in the early stages of stress treatment but increased in the later stages, and this effect was relieved in the later stages. These results suggest that SA participates in the salt tolerance response of *M. tricuspidata.*

### 3.2. Phenylpropane and Flavonoid Biosynthesis Pathways Involved in the Response to Salt Stress

Our transcriptomic analysis revealed that the majority of genes involved in phenylpropane biosynthesis were significantly upregulated during the early stage of salt treatment (2 and 24 h) (Figure 7A). However, in the later stage (48 h), the expression level of *4CL* in the general phenylpropane biosynthetic pathway, as well as key genes in the specific lignin biosynthetic pathway, such as *CCR*, *CAD*, and *CSE*, were significantly upregulated. This suggests that lignin synthesis was not inhibited under salt stress conditions. Cinnamates and flavonoids in the mulberry ‘Guisangyou 12′ were induced and expressed under salt stress, and studies have shown that phenylpropane may be involved in salt stress response and enhance mulberry’s salt tolerance [54]. Lignin plays a protective role in cells against abiotic stresses and serves as a structural component that helps perceive and respond to environmental stresses [55,56]. The upregulation of these genes may protect plants from salt stress, and the upregulation of lignin biosynthesis could be an important defense mechanism in *M. tricuspidata* against salt stress.

The present study identified DEGs in the flavonoid biosynthesis pathway that were upregulated and downregulated in the first and last comparison groups, respectively (Figure 7B), exhibiting an opposite trend under different stress times. This is consistent with previous research on *Belamcanda chinensis*, where the levels of flavonoids varied with the degree and duration of drought stress [57]. Zhang et al. found that, as salt stress increased, the flavonoid content in *Cyclocarya paliurus* also showed an increasing trend [58]. Under single and combined stress of NaCl and CdCl_2_, flavonoids were found to be involved in maintaining the normal state of *Tamarix hispida* [59]. Research on *Glycyrrhiza uralensis* has found that *PAL*, cinnamate 4-hydroxylase (*C4H*), *4CL*, *CHS*, chalcone-isomerase (*CHI*), and *FLS* exhibited higher expression levels under salt treatment [60]. Salt treatment significantly increased the flavonoid biosynthesis pathway of *Cyclocarya paliurus* seedlings at the metabolic level to cope with salt stress [61]. Consistently, Wanichthanarak et al. found that the KEGG pathways were enriched in plant hormone signal transduction, phenylpropanoid, and flavonoid biosynthesis in rice to respond to salinity stress [62]. Therefore, our transcriptomic analysis revealed significant changes in genes related to flavonoid biosynthesis, and the dynamic changes in flavonoids may play a role in alleviating salt stress in *M. tricuspidata*.

### 3.3. Alkaloids and Plant MAPK Signaling Pathway Enhance the Salt Tolerance

Alkaloids, as secondary metabolites of tryptophan and glutamic acid synthesis, are also involved in defense against salt stress [63]. In this study, the tropane, piperidine, and pyridine alkaloid biosynthesis pathway revealed that four important genes, namely *TAT*, *TR1*, *AOC3*, and *GOT1*/*2*, were upregulated in most of the comparison groups (Figure 7C). Especially under 48 h treatment, the upregulated expression of *AOC3* and *GOT1*/*2* was more pronounced, suggesting that the accumulation of these genes may be a defensive response to salt stress. Jia et al. found that the changes in alkaloid levels in *Malus halliana* can alleviate the damage caused by ROS, thereby enhancing its resistance to salt stress [64]. Triple mutants of *Atahp2,3,5* and *Atarr1,10,12* in *Arabidopsis* exhibited greater salt tolerance, and transcriptomic analysis revealed that the DEGs were mainly enriched in phenylpropanoid, flavonoid, terpenoid, piperidine, and pyridine alkaloid biosynthesis, as well as plant hormone signaling transduction [65].

MAPK is generally considered to play a crucial role in the biological response to environmental stress by phosphorylating downstream signaling pathways [66]. In this study, four types of MAPK signaling transduction genes were identified, including *MKK9*, *MPK3*, and *MAPKKK17*/*18*. The early stage (2 h) of salt treatment induced upregulated expression of *MKK9* and *MPK3*, while the expression of *MAPKKK17*/*18* was significantly upregulated in the later stage (48 h) (Figure 7D). Yan et al. reported that *MPK3*/*6* interact with each other and phosphorylate the key cytokinin signaling components of *ARR1*/*10*/*12* under salt stress [67]. Additionally, *OsMAPKKK63* (*Arabidopsis* ortholog *AtMPKKK15*/*16*/*17*/*18*) was induced by high salt and interacted with *OsMKK1*/*6*, the mediators of salt stress response, and mutants of *OsMAPKKK63* indeed demonstrated the necessity of the normal response to high salt in rice [68]. The genome-wide identification of MAPK has revealed its potential in improving salt tolerance in *Gossypium hirsutum* [69]. MAPK signal transduction may induce a large number of antioxidants and free radical scavenging enzymes in *Dendrobium huoshanense*, thereby enhancing its salt tolerance [70]. Therefore, we speculate that the MAPK signaling pathway may play a vital role in enhancing the salt tolerance of *M. tricuspidata* in response to salt stress.

### 3.4. Amino Acid Metabolism Plays a Role in Enhancing the Salt Tolerance of M. tricuspidata

Amino acids accumulate in response to various abiotic stress tolerances and serve a variety of functions in plant growth and development [71]. The present study’s metabolomic analysis revealed that the DAMs under salt treatments were mainly associated with alanine, aspartate, and glutamate metabolism, as well as the glycine, serine, and threonine metabolism pathways (Figure 8B). Research on *M. halliana* has found that the upregulation of the MhASP3 protein involved in the synthesis of aspartate and glutamate initiates amino acid defense responses to counteract salt stress [64]. The integration analysis of transcriptome and metabolome under salt stress revealed a strong enhancement of amino acid metabolism, including alanine, aspartate, glutamate, glycine, serine, and threonine, contributing to salt tolerance in *Sesamum indicum* [72]. A metabolomic analysis of *Glycine max* revealed that salt-tolerant soybean enhances salt tolerance by enhancing amino acid and organic acid metabolism [73]. Moreover, research has found that the accumulation of several amino acids, including serine, phenylalanine, and threonine, can enhance plant salt tolerance by mediating the ROS clearance and controlling damage repair processes [74,75]. The above metabolites were also present in our study, indicating that amino acid metabolism plays an essential role in the salt tolerance response in *M. tricuspidata*. Notably, in our integrated analysis, we also identified glycine, serine, and threonine metabolism as an important one that showed a significant correlation, indicating its crucial role in the salt stress response process. However, the butanoate metabolism and 2-oxocarboxylic acid metabolism pathways were unique in this study, suggesting that their presence indicates different mechanisms of salt tolerance in different plants.

## 4. Materials and Methods

### 4.1. Plant Material

*M. tricuspidata* seedings were collected from the Dafeng provenance in Yancheng, Jiangsu province (120°85′ E; 32°98′ N). These seedlings were cultivated in the greenhouse of Jiangsu Academy of Forestry (118°46′ E; 31°51′ N) for a growth period of 150 d, serving as the plant materials for this study. Under natural outdoor conditions, healthy seedlings were cultivated in culture containers filled with a mixture of peat soil and perlite in a ratio of 3:1 (*v*/*v*). The cultivation container used was a black plastic box measuring 500 cm × 450 cm × 380 cm.

### 4.2. Salt Treatment and Sample Collection

The seedlings were treated as follows: (a) control (CK: 0 mM NaCl); (b) low concentration (l: 34 mM NaCl); (c) moderate concentration (m: 68 mM NaCl); and (d) high concentration (h: 102 mM NaCl). These concentration levels were selected and adjusted based on previous research on salt tolerance in plants of the Moraceae family [76]. Meanwhile, four different time points (2, 24, 48, and 72 h) were chosen to observe the physiological and molecular responses to salt stress. Each treatment had 3 biological repeats, with each replicate consisting of 9 plants, resulting in a total of 108 plants (3 × 4 × 9). On September 26th, 2022, a 2 L NaCl solution of the corresponding concentration was irrigated once into each culture container, while the control treatment received an equal volume of pure water. Container trays were used to collect any exudate, which was then poured back into the container to prevent any outflow.

After each time point of the stress treatments, three samples were collected. Each sample consisted of three randomly selected plants combined to create a mixed sample. Each of these mixed samples contained 27 functional leaves from the same part of each plant, all with the same age. This provides the necessary tissue for the 3 replicates of physiological characteristics and transcriptome analysis, as well as 6 replicates for metabolomics. The collected samples were deposited in a −80 °C refrigerator until further use.

### 4.3. Physiological Characteristics Measurements

Plant soluble sugar (Product model: A145-1-1) and malondialdehyde (MDA) (Colorimetric method) (A003-3-1) contents, CAT (Visible light) (A007-1-1) and POD (A084-3-1) activities, total SOD (Hydroxylamine method) (A001-1-2), and the chlorophyll a and chlorophyll b (A147-1-1) concentrations were determined following the instructions provided in the kits obtained from Nanjing Jiancheng Bioengineering Institute (Nanjing, Jiangsu, China). Plant-soluble sugars, enzyme activities, and chlorophyll were extracted by homogenizing plant leaf tissue.

### 4.4. Transcriptome Analysis

#### 4.4.1. RNA Extraction and Library Construction

Total RNA was extracted using a FastPure plant total RNA isolation kit (Polysaccharides & Polyphenolics-rich) from VazymeBiotech, Nanjing, China, following the manufacturer’s protocol. The quality, concentration, and integrity of RNA were assessed using NanoPhotometer spectrophotometer (Implen, Calabasas, CA, USA), Qubit2.0 fluorometer (Invitrogen, Carlsbad, CA, USA), and Agilent 2100 bioanalyzer (Agilent Technologies, Palo Alto, CA, USA), respectively. RNA-seq libraries were generated using the NEBNext^®^ Ultra^TM^ RNA Library Prep Kit for Illumina^®^ NEB, #E7530L, Ipswich, MA, USA) according to the manufacturer’s instruction, and then sequenced using an Illumina NovaSeq6000 (Illumina, San Diego, CA, USA).

#### 4.4.2. Data Analysis and Functional Annotation 

The sequencing data were filtered using Fastp (version 0.23.2) (https://github.com/OpenGene/fastp, accessed on 6 September 2022) [77]. After filtering out reads containing adapter sequences, over 10% N bases, all A bases, and low-quality (Q ≤ 20) readings of more than 50 bases, the resulting clean reads were obtained. The cleaned reads were then de novo assembled into transcripts using Trinity software (version 2.13.2) (https://github.com/trinityrnaseq/trinityrnaseq/, accessed on 10 September 2022) [78]. The integrity of the assembly sequence was assessed using BUSCO software (version 3.0.2) [79]. The expression abundance of the unigenes was calculated using RSEM software (version 3.0.2) in terms of fragments per kilobase of exon per million mapped reads (FPKM) values [80]. DEGs were identified using the DESeq2 software (version 1.6.3) based on the criteria of false detection rate (FDR) < 0.05 and (|log2 fold change (FC)| > 1) [81]. The DEGs were functionally annotated using the Gene Ontology (GO) and Kyoto Encyclopedia of Genes and Genomes (KEGG) pathways [82]. The significantly enriched GO terms and KEGG pathways were determined based on an FDR cutoff of 0.05.

### 4.5. Metabolomics Analysis

#### 4.5.1. Metabolites Extraction and Ultra-High-Performance Liquid Chromatography (UHPLC)-MS/MS Analyses

The ground leaf tissue samples (100 mg) were placed in an Eppendorf (EP) tube and resuspended in 500 μL of prechilled 80% methanol. The mixture was incubated in an ice water bath for 5 min, followed by centrifugation at 4 °C, 15,000× *g* for 20 min. Subsequently, a portion of the supernatant was diluted to a final concentration of 53% methanol. The diluted samples were transferred to new EP tube and centrifuged again. Finally, the supernatant was injected for analysis using an LC-MS/MS system [83].

The UHPLC-MS/MS analysis was performed using the Vanquish UHPLC system (Thermo Fisher, Dreieich, Germany) connected to the Orbitrap Q ExactiveTM HF-X mass spectrometer (Thermo Fisher, Dreieich, Germany). The samples were injected onto a Hypesil Gold column (100 × 2.1 mm, 1.9 μm) and eluted with a linear gradient at a flow rate of 0.2 mL/min for 17 min. For the positive polarity mode, the eluent A consisted of 0.1% formic acid in water, while the eluent B was methanol. For the negative polarity mode, the eluent A was 5 mM ammonium acetate (pH = 9.0), and the eluent B was methanol. The solvent gradient settings were as follows: 2% B for 1.5 min; 2–100% B for 12 min; 100% B for 14 min; 100–2% B for 14.1 min; and 2% B for 17 min. During the analysis, the spray voltage was 3.2 kV, the capillary temperature was 320 °C, and the flow rate of the sheath gas and aux gas were 40 Arb and 10 Arb, respectively.

#### 4.5.2. Data Processing, Metabolite Identification, and Multivariate Statistical Analysis

Compound discoverer 3. 1 (CD3.1, Thermo Fisher, Hercules, CA, USA) was used to process the raw data files generated by UHPLC-MS/MS, and peak alignment, peak picking, and quantitation were performed for each metabolite. The main characteristics were as follows: retention time tolerance, 0.2 min; actual mass tolerance, 5 ppm; signal intensity tolerance, 30%; signal/noise ratio, 3; and minimum intensity threshold, 100,000. Afterwards, the peak intensities were normalized to the total spectral intensity. Molecular formulas were predicted using the normalized data of additive ions, molecular ion peaks, and fragment ions. The obtained peaks were matched with the mzCloud (https://www.mzcloud.org/, accessed on 12 January 2023). Accurate qualitative and relative quantitative results were obtained from mz Vaultand Mass Listdatabase. Quality control samples were used to assess the system’s stability, and blank samples were employed to remove the background ions. Statistical analysis was performed using R software (version 3.4.3), Python (version 2.7.6) and CentOS (version 6.6). 

#### 4.5.3. Identification of Differential Metabolites and Analysis of Metabolic Pathway

The variable importance in the projection (VIP) scores of orthogonal partial least squares discriminant analysis (OPLS-DA) and the *p*-values from *t*-tests were utilized to identify DAMs [84]. Metabolites were defined as differential whenever VIP ≥ 1 and *p*-value < 0.05, and were mapped to the KEGG metabolic pathways for annotation and enrichment analysis.

### 4.6. Integrative Analysis of Omics Data

In the association analysis, a shared KEGG pathway analysis was performed to identify a common pathway involving differential genes and metabolites between groups [85]. For the integrative analysis, a Pearson correlation coefficient model was employed. The correlation was established by calculating the expression of all differential genes and the abundance of all differential metabolites using a union approach across the comparison groups.

### 4.7. Statistical Analysis

IBM SPSS statistics (version 21) (IBM Inc., Armonk, NY, USA) was used to perform the analysis of variance (ANOVA), and significant variations were determined using Duncan’s multiple range test method at a significance level of *p* = 0.05. The Cytoscape program (version 3.3.0) (NIGMS, Bethesda, MD, USA) was used to perform the network regulation diagram.

### 4.8. Quantitative Real-Time PCR (qRT-PCR) Validation of DEGs

qRT-PCR analysis was used to verify the accuracy of transcriptome profiles. The gene expression levels of 9 randomly selected genes were analyzed. Relative expression levels were calculated by the 2^−ΔΔCt^ method [86]. Ubiquitin (UBQ) was selected as an internal reference, and PCR primers for the tested genes were designed based on the RNA-Seq data (Supplementary Table S4).

## 5. Conclusions

**Figure 10:**
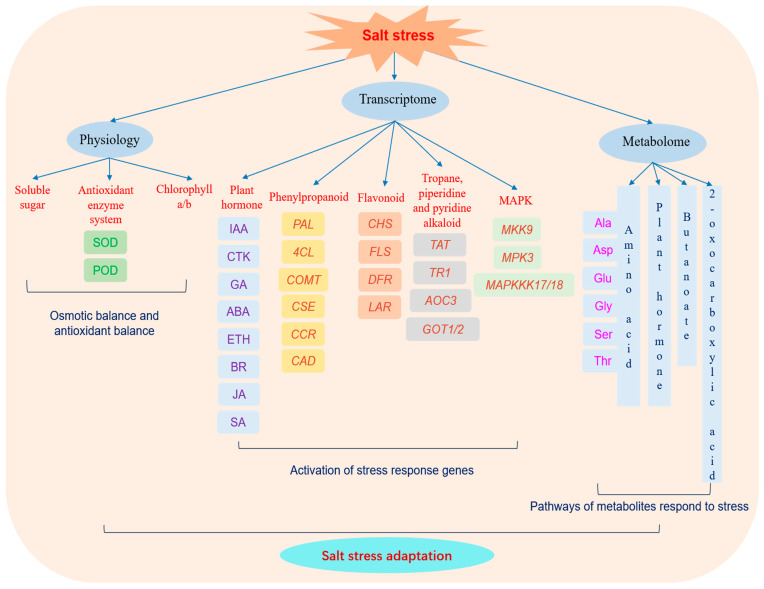
Schematic diagram of the mechanism of salt stress adaptation in *M. tricuspidate*.

In the current study, we conducted an integrated analysis of physiology, transcriptome, and metabolome in *M. tricuspidata* under salt treatment. Our findings revealed that the plant’s salt adaptability is associated with maintaining appropriate levels of soluble sugar, increased activities of SOD and POD, as well as the maintained concentrations of chlorophyll a and chlorophyll b. Through KEGG pathway enrichment analysis, we identified key DEGs directly involved in plant hormone signal transduction, phenylpropanoid and flavonoid biosynthesis, alkaloids, and the MAPK signaling pathway. Furthermore, our differential metabolite analysis unveiled the involvement of the main pathways related to amino acid metabolism (alanine, aspartate and glutamate; glycine, serine, and threonine), butanoate and 2-oxocarboxylic acid metabolism, and the biosynthesis of plant hormones. Notably, the glycine, serine, and threonine metabolism pathway showed significance in both the metabolome and transcriptome–metabolome correlation analyses, highlighting its crucial role in response to salinity. Based on our findings, we constructed a regulatory mechanism model depicting salt stress adaptation in *M. tricuspidata* (Figure 10). This study will contribute to our understanding of the molecular mechanism underlying salt tolerance in *M. tricuspidata*, and provide valuable insights for the future development of salt-tolerant breeding and the improvement in salt land.

## Figures and Tables

**Table 1:** The number of DAMs between the different comparison groups.

Ion Mode	Group	Up	Down	Total
POS	CK2-vs.-m2	18	38	56
	CK2-vs.-h2	57	59	116
	CK24-vs.-m24	4	16	20
	CK24-vs.-h24	54	36	90
	CK48-vs.-m48	44	35	79
	CK48-vs.-h48	39	36	75
NEG	CK2-vs.-m2	48	65	113
	CK2-vs.-h2	70	63	133
	CK24-vs.-m24	10	31	41
	CK24-vs.-h24	32	44	76
	CK48-vs.-m48	44	29	73
	CK48-vs.-h48	26	32	58

## Data Availability

The transcriptome sequence data for *Maclura tricuspidate* were deposited at NCBI under BioProject code PRJNA1065092. The original contributions presented in the study are included in the article/Supplementary Materials.

## Supplementary Materials

The following supporting information can be downloaded at: https://www.mdpi.com/article/10.3390/plants13030397/s1. Figure S1: Overview of *M. tricuspidate* transcriptome responses to salt stress. (A) Principal component analysis (PCA) plots of transcripts under salt stress. (B) The Pearson correlation coefficient between the samples shown in the heat map. Table S1: Summary of the RNA sequencing data. Table S2: Information of metabolites involved in metabolic pathways. Table S3: Information of metabolites involved in glycine, serine, and threonine metabolism. Table S4: Primers used in qRT-PCR analyses.
