# Supplementary figures and images for: Integration of Physiological, Transcriptomic, and Metabolomic Analyses Reveal Molecular Mechanisms of Salt Stress in Maclura tricuspidata
Source: Plants (Basel). 2024 Jan 29;13(3):397. doi: 10.3390/plants13030397 (PMC10857159; doi:10.3390/plants13030397)

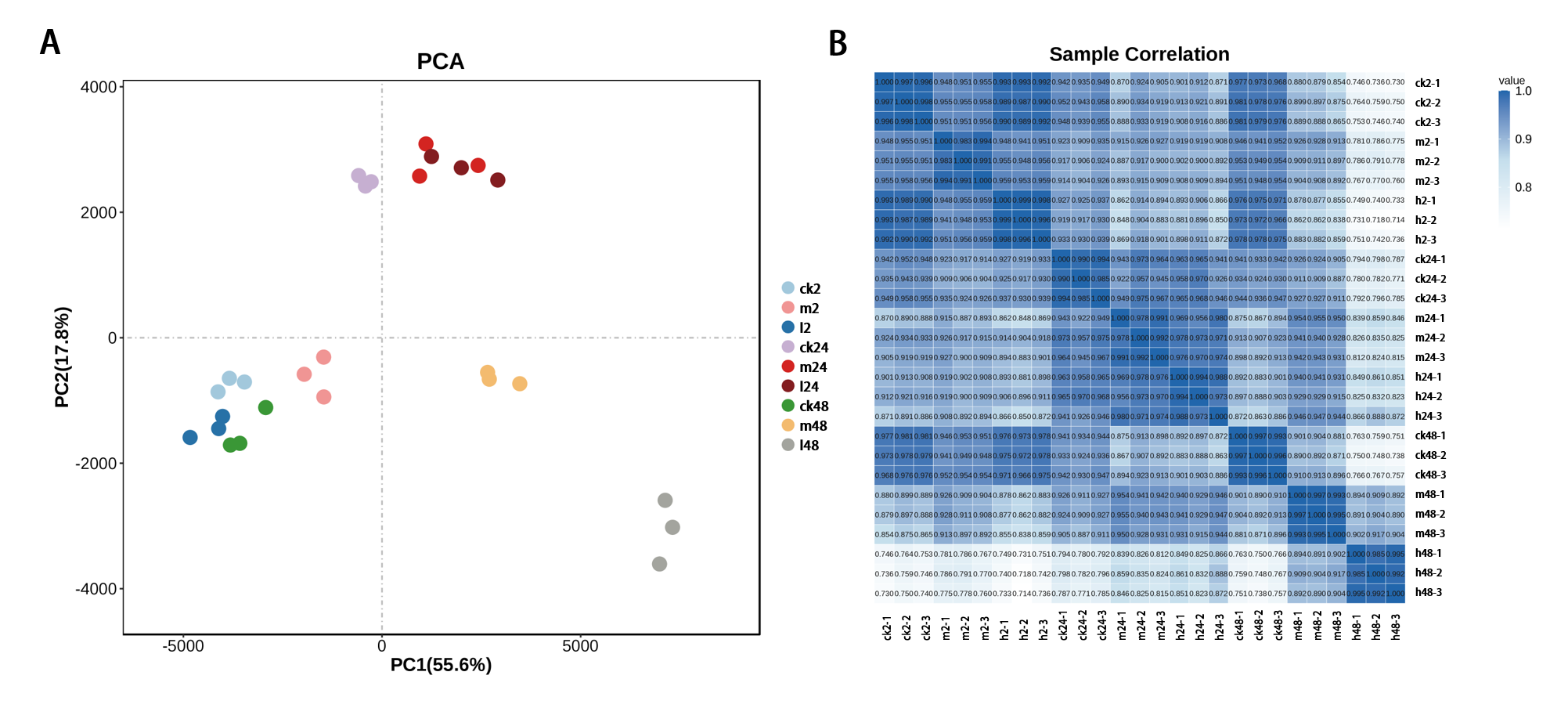

Supplement: Supplementary file 1 [file plants-13-00397-s001.zip › plants-2816475-supplementary/Supplementary Figure S1.tif]
